# Low TIP30 Protein Expression is Associated with a High Risk of Metastasis and Poor Prognosis for Non-Small-Cell Lung Cancer

**DOI:** 10.3390/jcm8010083

**Published:** 2019-01-12

**Authors:** Chao-Ju Chen, Po-An Chou, Ming-Shyan Huang, Yu-Peng Liu

**Affiliations:** 1 Graduate Institute of Medicine, College of Medicine, Kaohsiung Medical University, Kaohsiung 807, Taiwan; u102509001@kmu.edu.tw or 990321@kmuh.org.tw; 2 Department of Laboratory Medicine, Kaohsiung Medical University Hospital, Kaohsiung Medical University, Kaohsiung 807, Taiwan; 3 Division of Pulmonary and Critical Care Medicine, Department of Internal Medicine, E-DA Cancer Hospital, Kaohsiung 807, Taiwan; realdfs@livemail.tw (P.-A.C.); 770261@gmail.com (M.-S.H.); 4 School of Medicine, College of Medicine, I-Shou University, Kaohsiung 807, Taiwan; 5 Graduate Institute of Clinical Medicine, College of Medicine, Kaohsiung Medical University, Kaohsiung 807, Taiwan; 6 Research Center for Environmental Medicine, Kaohsiung Medical University, Kaohsiung 807, Taiwan; 7 Center for Infectious Disease and Cancer Research, Kaohsiung Medical University, Kaohsiung 807, Taiwan

**Keywords:** non-small-cell lung cancer (NSCLC), Tat-interacting protein 30 (TIP30), biomarker, precision medicine

## Abstract

Non-small-cell lung cancer (NSCLC) is a deadly malignancy with a high prevalence worldwide. A reliable biomarker that can predict the prognosis is required to determine the therapeutic strategy. TIP30 was first identified as a tumor suppressor. A number of mechanistic studies indicated that the downregulation of TIP30 enhances the stemness, migration and survival of NSCLC cells. However, the clinical relevance of TIP30 for the prognosis of NSCLC is unknown. From a meta-analysis of public microarray datasets, we showed the upregulation of TIP30 mRNA expression was associated with worse overall survival of NSCLC patients, which contradicted the tumor suppressive role of TIP30. It is worth noting that the TIP30 mRNA expression was not correlated with its protein expression in 15 NSCLC cell lines. The results from the immunohistochemistry of a tissue microarray showed the downregulation of the TIP30 protein expression was associated with a higher risk of metastasis. In addition, the decrease in TIP30 protein was correlated with worse overall and progression-free survival of the NSCLC patients. Multivariate analysis suggested the loss of TIP30 protein was an independent factor to predict the poor prognosis of NSCLC. Our data indicated that TIP30 protein, not mRNA, would be a potential prognostic biomarker of NSCLC.

## 1. Introduction

Lung cancer is the most common cause of cancer-related mortality worldwide, and non-small-cell lung cancer (NSCLC) accounts for nearly 80% of these cases [1,2]. Despite advances in the diagnosis and treatment of NSCLC in recent decades, the prognosis for patients with recurrent or advanced lung cancer remains poor [3,4]. Therefore, a better understanding of the pathogenesis of NSCLC progression and the identification of valuable prognostic biomarkers to classify the relapse risk for NSCLC patients are eagerly desired.

The 30-kDa Tat-interacting protein (TIP30), also known as HTATIP2 or CC3, was initially identified as a tumor suppressor and is involved in many biological events at various stages of tumor progression, such as tumor initiation, cell proliferation, angiogenesis, metastasis and chemoresistance [5,6,7,8]. TIP30 is frequently downregulated in different cancer types, including hepatocellular carcinoma, colorectal carcinoma, breast cancer, gastric cancer, prostate cancer and lung cancer [4,9,10,11,12,13]. The deletion of TIP30 leads to the spontaneous development of lung cancer in mice [14]. The loss function of TIP30 can be regulated by genetic or epigenetic mechanisms and promotes the activation of signaling pathways that contribute to cell proliferation, survival and mobility [15,16,17]. 

The possible mechanisms of TIP30-regulated disease progression and metastasis have been demonstrated in cell and animal models [18]. TIP30 may inhibit tumor growth through the suppression of cyclin D1 transcription [5]. The decrease of TIP30 expression promotes the nuclear translocation of Snail, which leads to an epithelial–mesenchymal transition and the distant metastasis of lung cancer cells [19]. In addition, the reduction of TIP30 potentiates lipid metabolism and the proliferation of hepatocellular carcinoma cells [20]. Accordingly, TIP30 has been considered as a biomarker to predict the therapeutic outcome of cancer patients [21,22].

Genome-wide cDNA microarray and high-throughput sequencing have been widely used to generate extensive gene expression profiles of tumors in different cancer types. By using these mRNA expression-based approaches, a large number of prognostic biomarkers or signatures in lung cancer have been suggested, but most of them have minimal routine clinical application [23,24,25,26,27,28]. Some biomarkers were discovered according to their clinical relevance, but there was a lack of rigorous experimental evidence to demonstrate the cause-and-effect relationships. On the other hand, the association between biomarker levels and disease progression is not consistent in different cohorts, which may be due to the small number of cases or different probes used in the assay. In view of these limitations, a more comprehensive and in-depth understanding of biomarkers in terms of their biological process and clinical significance is necessary.

Although TIP30 has been considered a prognostic biomarker for some cancer types, the association between TIP30 expression and disease progression in NSCLC is unknown. It has been shown that the TIP30 protein expression was inversely correlated with lymph node metastasis in lung adenocarcinoma patients [29]. Thus, we hypothesized that the TIP30 expression level may predict the prognosis of NSCLC patients. We performed a meta-analysis of the publicly available microarray datasets to investigate the relationship between the TIP30 mRNA expression level and the clinical prognosis in NSCLC patients. In addition, we studied the correlation of the TIP30 protein expression level with the clinicopathological features in a tissue microarray, which consisted of tumor specimens from 113 NSCLC patients. Our results suggested that the TIP30 protein expression level, but not mRNA expression level, was negatively correlated with the progression-free and overall survival of NSCLC patients.

## 2. Materials and Methods

### 2.1. Data Extraction from PrognoScan Database and Methodological Assessment

The associations between the TIP30 mRNA expression levels and the prognosis of NSCLC patients were obtained from the PrognoScan database. PrognoScan is a large collection of publicly available cancer microarray datasets with clinical information, and is also a tool for evaluating the relationship between gene expression and prognosis [30]. It is publicly accessible at http://dna00.bio.kyutech.ac.jp/PrognoScan/ [31]. Human TIP30 (HTATIP2) gene was input as a query and the data were collected for meta-analysis. The meta-analysis was performed using the RevMan 5 software provided by the Cochrane Collaboration, available at: https://community.cochrane.org/help/tools-and-software/revman-5/revman-5-download [32]. We used the hazard ratio (HR) value to evaluate the relationship between the TIP30 mRNA level and the relapse-free or overall survival of the patients. *P* < 0.05 was considered as a significant difference. The I^2^ test was used to examine the heterogeneity between each study.

### 2.2. Cell Culture

The human squamous cell carcinoma cell lines, H157 and H520, adenocarcinoma cell lines H358, H441, H928, H1355, PC9, PC14, CL1-0 and CL1-5 and large-cell carcinoma cell lines H460, H661, H1299 and PC13 were cultured in Roswell Park Memorial Institute (RPMI) 1640 medium supplemented with 10% fetal bovine serum, 100 U/mL penicillin and 100 μg/mL streptomycin (Gibco, Grand Island, NY, USA). The human adenocarcinoma cell line, A549, was cultured in Dulbecco’s modified Eagle medium (DMEM). The medium was supplemented with 10% fetal bovine serum, 100 U/mL penicillin and 100 μg/mL streptomycin (Gibco). All the cell lines were incubated in 5% CO_2_ in a 37 °C incubator. All cell lines were confirmed to be mycoplasma-negative by DNA staining. The human NSCLC cell lines A549, H157, H358, H441, H460, H520, H661, H1299, H1355 were purchased from American Type Culture Collection (Manassas, VA, USA). The H928, PC9, PC13, PC14, CL1-0, CL1-5 cell lines were a kind gift from Dr. Michael Hsiao (Genomics Research Center, Academia Sinica, Taipei, Taiwan).

### 2.3. RNA Isolation and Quantitative Real-Time Polymerase Chain Reaction (qPCR)

The total RNA was extracted with TRIzol (Thermo Fisher Scientific, Waltham, MA, USA) according to the manufacturer’s instructions. RNA was then reverse transcribed into cDNA with the M-MLV Reverse Transcriptase Kit (Thermo Fisher Scientific). The quantitative real-time polymerase chain reaction (qPCR) was set up using Fast SYBR Green Master Mix (Thermo Fisher Scientific) on a Step-One Plus real-time PCR system (Thermo Fisher Scientific) for 20 s at 95 °C, followed by 40 cycles at 95 °C for 3 s and annealing at 60 °C for 20 s. The results were normalized to the housekeeping gene glyceraldehyde 3-phosphate dehydrogenase (GAPDH).

### 2.4. Western Blot Analysis

The cells were harvested and lysed in RIPA buffer containing a protease inhibitor cocktail (Roche, Mannheim, Germany). The protein concentrations were determined using a Bio-Rad DC protein assay kit (Bio-Rad, Hercules, CA, USA). Subsequently, 20 μg of total protein was loaded onto a 10% sodium dodecyl sulfate (SDS)-polyacrylamide gel for electrophoresis and transferred to a polyvinylidene difluoride (PVDF) membrane. The protein was identified by incubating the membrane with primary antibodies followed by horseradish peroxidase-conjugated secondary antibodies and an enhanced chemiluminescence solution (NEN Life Science, Boston, MA, USA). The following antibodies were used in this study: anti-TIP30 (Genetex, Irvine, CA, USA) and anti-Actin (Sigma, St. Louis, MO, USA).

### 2.5. Tissue Microarray

The tissue microarray (TMA-38AB) consisted of the tumor samples from 113 NSCLC patients enrolled in Kaohsiung Medical University Hospital from 1991 to 2007, with the Institutional Review Board’s approval (KMUH-IRB-2011-0286). The histologic diagnosis of the specific type of lung cancer was made according to the recommendations of the World Health Organization (WHO) classification, and the tumor size, local invasion, lymph node involvement, distal metastasis and final disease stage were determined according to the 7th edition of the tumor, node, metastasis (TNM) staging system for lung cancer by the International Union Against Cancer and the American Joint Committee on Cancer.

### 2.6. Immunohistochemistry (IHC) and Patient Survival Analysis

The tissue sections or the tissue microarray samples (5 μm) were dewaxed and rehydrated. Antigen retrieval was performed by incubating the slides in 10 mM sodium citrate buffer (pH = 6.0) and microwaving the samples for 20 min. After blocking with 3% H_2_O_2_ and 10% normal goat serum, the slides were incubated with rabbit polyclonal antibodies against TIP30 (1:400) at 4 °C overnight. The slides were then incubated with a biotin-conjugated anti-rabbit secondary antibody followed by an incubation with the polymer-horseradish peroxidase (HRP) reagent, using the ABC kit from Vector Laboratories (Burlingame, CA, USA). The peroxidase activity was visualized with a diaminobenzidine tetrahydroxychloride solution (Vector Laboratories). The sections were counterstained with hematoxylin.

For the immunohistochemistry (IHC) staining interpretation, the intensity of the staining signal was defined as follows: 0, no staining; 1, weak staining; 2, moderate staining; and 3, strong staining. All the patients were divided into two groups according to the IHC scores. The tumor samples with scores of 0 and 1 were defined as low IHC expression level and scores of 2 and 3 were defined as high expression. The survival curves were analyzed using the Kaplan–Meier method, and the Cox proportional hazard regression was used to examine the prognostic significance of the factors in the univariate and multivariate models. All the statistical tests were two-sided. *P* < 0.05 was considered significant. The analyses were performed using the SPSS (Statistical Package for the Social Sciences, version 13.0) software.

## 3. Results

### 3.1. Meta-Analysis Revealed Poor Association between TIP30 mRNA Expression and the Prognosis of Lung Cancer Patients

**Figure 1:**
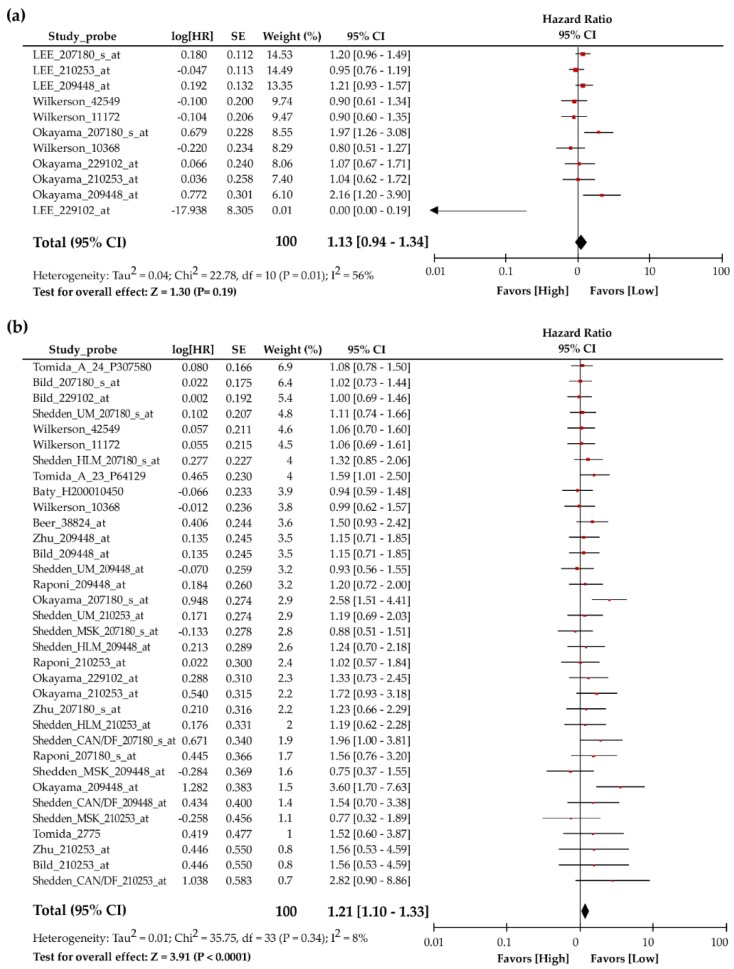
Association between TIP30 mRNA expression and the survival of non-small-cell lung cancer (NSCLC) patients. The horizontal bars in the forest plot represent the 95% confidence intervals of the hazard ratio of relapse-free survival. (**a**) Three publicly available microarray datasets encompassing 398 NSCLC patients were used for a meta-analysis of the association between TIP30 mRNA expression and relapse-free survival. (**b**) The meta-analysis of the TIP30 mRNA expression for the overall survival of NSCLC was performed by using 9 published microarray datasets encompassing 1325 NSCLC patients.

To evaluate whether the TIP30 expression level could be a biomarker for the disease progression of lung cancer, we first studied the correlation of TIP30 mRNA levels and the prognosis of lung cancer patients from the publicly available microarray datasets in the PrognoScan database. Among different cohorts and probes, heterogeneous results for the hazard ratio in relapse-free survival (Table 1) and overall survival (Table 2) were obtained. To confirm the clinicopathological role of the TIP30 mRNA expression level in NSCLC patients, we performed a meta-analysis using 3 microarray datasets with 11 probes for the relapse-free survival of lung cancer patients. The hazard ratio values for relapse-free survival were not significantly different between high TIP30 mRNA expression patients and low TIP30 mRNA expression patients (HR = 1.13, 95% CI = 0.94–1.34, *P* = 0.19, I^2^ = 56%) (Figure 1a). Likewise, the data for the TIP30 mRNA expression were extracted from 9 microarray datasets with 34 probes for the meta-analysis of overall survival (Table 2). The results revealed that the NSCLC patients with higher TIP30 mRNA expression showed worse overall survival than those with lower TIP30 mRNA expression (HR = 1.13, 95% CI = 1.10–1.33, *P* < 0.0001, I^2^ = 8%) (Figure 1b). These results were not consistent with previous findings showing that TIP30 functioned as a tumor suppressor and inhibited metastasis.

### 3.2. The TIP30 Protein Expression Level is Not Determined by the mRNA Level in Human Lung Cancer Cell Lines

**Figure 2:**
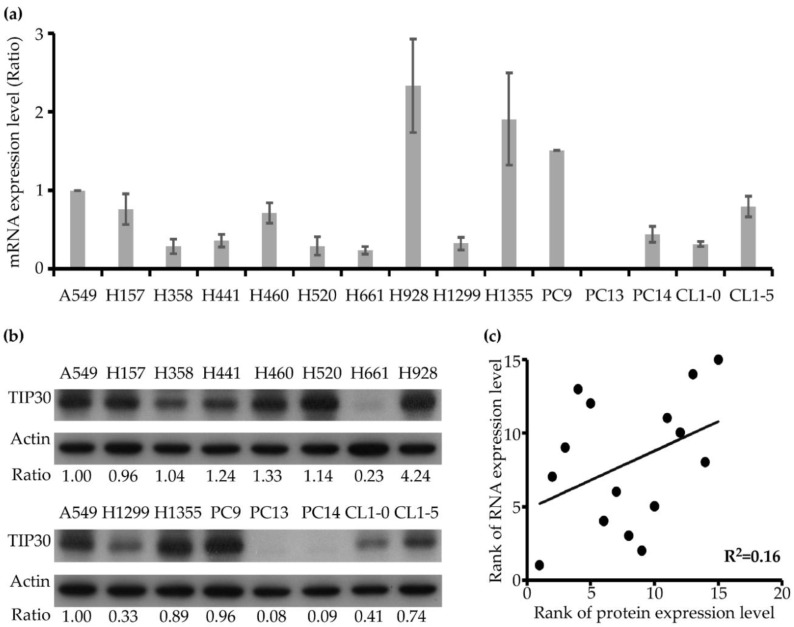
Comparison of the TIP30 mRNA and protein expression levels in the 15 NSCLC cell lines. (**a**) The TIP30 mRNA expression in the 15 NSCLC cell lines was determined by quantitative real-time polymerase chain reaction (qPCR). The data was normalized to the TIP30 mRNA level in the A549 cell line. (**b**) The TIP30 protein expression in the 15 NSCLC cell lines was determined by Western blotting. The pixel intensities of the blotting signals corresponding to individual cell lines were normalized to the signal intensity of the A549 cell line. (**c**) The TIP30 mRNA and protein expression levels of the 15 lung cancer cell lines were ranked and plotted. A linear regression model comparing the TIP30 protein (*x*-axis) and mRNA (*y*-axis) expression levels was performed. The coefficient of determination (R^2^) was indicated.

A previous study indicated that the protein expression of TIP30 could be regulated by miRNA-mediated epigenetic regulation [16]. Therefore, we hypothesized that the TIP30 mRNA expression level may not necessarily correlate with protein expression. Accordingly, we conducted a statistical comparison test between TIP30 mRNA and protein expression levels using a panel of 15 NSCLC cell lines, including squamous cell carcinomas (H157 and H520), adenocarcinomas (A549, H358, H441, H928, H1355, PC9, PC14, CL1-0 and CL1-5) and large-cell carcinomas (H460, H661, H1299 and PC13). We examined the TIP30 mRNA expression levels of the 15 NSCLC cell lines using qPCR and ranked them according to the mRNA expression level (Figure 2a). In parallel, we determined the TIP30 protein expression level of 15 NSCLC cell lines by Western blotting and also ranked them according to the protein expression level (Figure 2b). The ranking of the TIP30 mRNA expression in 15 cell lines (H928 > H1355 > PC9 > A549 > CL1-5 > H157 > H460 > PC14 > H441 > H1299 > CL1-0 > H358 > H520 > H661 > PC13) was not similar to that for the TIP30 protein expression level (H928 > H460 > H441 > H520 > H358 > A549 > H157 > PC9 > H1355 > CL1-5 > CL1-0 > H1299 > H661 > PC14 > PC13). Subsequently, we performed simple linear regression to determine the statistical correlation between the mRNA and protein expression level of TIP30. The result indicated that the correlation between the TIP30 mRNA and protein expression was weak (R^2^ = 0.16) (Figure 2c). This result suggested that the TIP30 protein expression may be regulated by a post-transcriptional mechanism in NSCLC cells. Indeed, it has been reported that miR-10b, a microRNA that directly targets TIP30, can induce cell proliferation and invasion by downregulating TIP30 expression [16,41].

### 3.3. Down-Regulation of the TIP30 Protein Correlates with Poor Clinical Outcomes in NSCLC Patients

**Figure 3:**
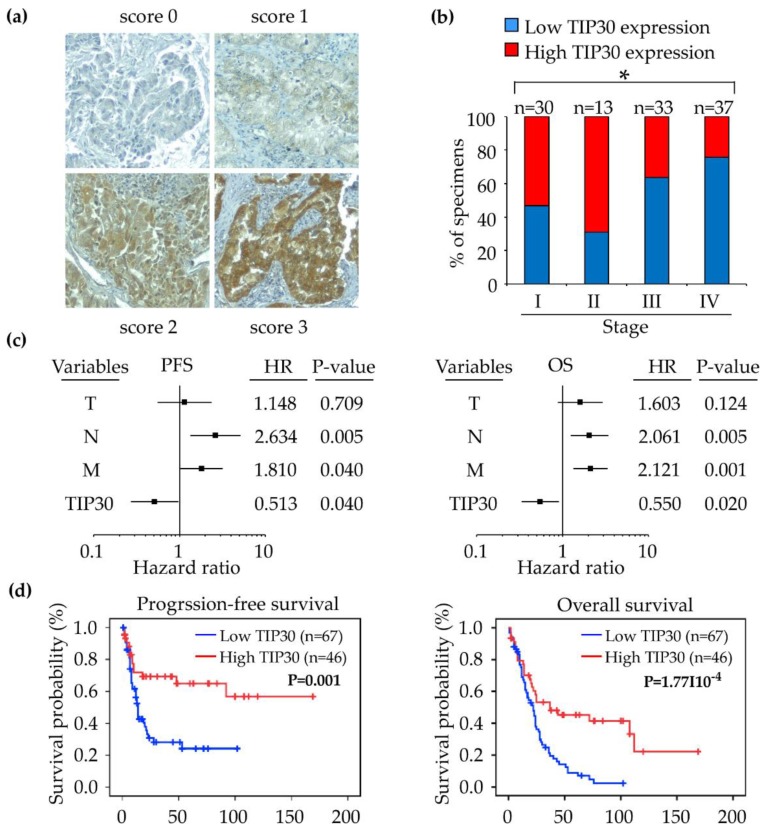
(**a**) The representative immunohistochemistry (IHC) images show the scoring of TIP30 expression from 0 to 3. (**b**) Quantification of TIP30 expression in the specimens from 113 NSCLC patients at different stages. * *P* < 0.05. (**c**) Forest plots from the multivariate Cox regression models of progression-free survival (PFS; left panel) and overall survival (OS; right panel). (**d**) Kaplan–Meier plots of the progression-free survival (left panel) and overall survival (right panel) of the 113 NSCLC patients, stratified by TIP30 levels.

Since the TIP30 mRNA expression level was not consistent with its protein level, we were interested in understanding whether TIP30 protein expression can be used as a biomarker to predict the prognosis of NSCLC patients. A tissue microarray, which consisted of clinical tumor samples from 43 patients with stage I–II NSCLC and 70 patients with stage III–IV NSCLC, was subjected to IHC to examine the protein expression levels of TIP30 in the tumors. Detailed clinicopathological information for all of the patients is provided in Table 3. The IHC staining signal of TIP30 was mainly distributed in the cytoplasm, but the nuclear location of TIP30 was also observed in some specimens. The staining intensity of TIP30 was scored on a scale from 0 to 3 (Figure 3a). According to the scoring criteria, the patients were classified into a TIP30-low group (scores 0 and 1) and a TIP30-high group (scores 2 and 3). The TIP30 expression levels were significantly correlated with advanced stages (stage III–IV; *P* = 0.013), distal metastasis (M; *P* = 0.027), histological types (*P* = 0.016), and recurrence (*P* = 0.002) (Figure 3b and Table 3).

To accomplish a prognostic evaluation, we analyzed the follow-up clinical data of the NSCLC patients who were enrolled in this cohort. Univariate and multivariate analysis revealed that the TIP30 protein expression level was a significant and independent predictor of the progression-free and overall survival of NSCLC patients (Figure 3c and Table 4). In addition, the Kaplan–Meier survival analysis revealed that a lower TIP30 protein expression level was significantly correlated with worse progression-free survival (*P* = 0.001) and overall survival (*P* < 0.001) compared to the patients with a high level of TIP30 protein expression (Figure 3d). The findings demonstrated that the TIP30 protein expression level is negatively correlated with the progression-free and overall survival of NSCLC patients. In other words, the TIP30 protein is a tumor suppressor and prognostic biomarker of NSCLC.

## 4. Discussion and Conclusions

In the era of precision medicine, the presence or absence of certain biomarkers or mutations may predict who may benefit from certain therapies and who may be a poor responder. An effective and reliable biomarker that can predict the prognosis of patients may benefit the therapeutic decision-making of physicians, and aid drug development. Since TIP30 was first identified as a tumor suppressor in hepatocellular carcinoma [42], it has been considered as a biomarker for a variety of malignancies [9,11,13,14,15,17,43,44,45]. However, the association between TIP30 and the prognosis of NSCLC patients remains unknown. In our study, we showed that TIP30 protein expression was inversely correlated with the distal metastasis and recurrence of NSCLC patients. This result is consistent with a previous study showing that the downregulation of TIP30 expression promoted metastasis in lung cancer [29]. Furthermore, high TIP30 protein levels were associated with the good prognosis of NSCLC patients. Multivariate regression indicated that the expression of the TIP30 protein was an independent factor able to predict the progression-free survival and overall survival of the NSCLC patients. These data supported the finding that the TIP30 protein level may be a potentially prognostic biomarker of NSCLC.

In our cohort, the lower protein levels of TIP30 significantly correlated with a high risk of recurrence and the distant metastasis of NSCLC. This result is not consistent with our meta-analysis of 3 publicly available microarray datasets, which showed that the TIP30 mRNA expression level was not significantly associated with the relapse-free survival of NSCLC patients. It is worth noting that the enrolled patients in these microarray-based datasets were American in GSE17710, Japanese in GSE31210, and Korean in GSE8894. Although TIP30 polymorphism in distinct populations is unknown, several TIP30 mutants have been identified in the tumors of hepatocellular carcinomas [15]. Interestingly, some TIP30 mutants lose their native tumor-suppressor activity and gain oncogenic activity [15]. The result of the meta-analysis may be masked by the different TIP30 genotypes in individual populations. For future studies, a classification of NSCLC patients with different TIP30 genotypes may be required.

A number of reports have demonstrated that the decreased TIP30 expression in metastatic tumors might be regulated by epigenetic mechanisms, including miRNAs and the hyper-methylation of the TIP30 promoter [16,17,41]. The epigenetic silencing of TIP30 by DNA hypermethylation was associated with a poor prognosis in hepatocellular carcinoma patients [17]. Besides, previous studies confirmed that TIP30 was a direct miR-10b target, and higher miR-10b levels were associated with poor prognosis in esophageal carcinoma and pancreatic cancer patients [16,41]. In the present study, we observed that the TIP30 mRNA expression level and the abundance of TIP30 proteins was not correlated in the 15 NSCLC cell lines. This result suggested that a post-transcriptional mechanism may be involved in TIP30 regulation in NSCLC cells and mRNA levels of TIP30 may not be a good biomarker for NSCLC patients. Comparatively, it is crucial to evaluate the association between the TIP30 protein expression level and the prognosis of NSCLC patients.

It has been shown that TIP30 harbors intrinsic dehydrogenases and kinase activity [46,47]. However, TIP30 may regulate tumor development in an enzyme-activity-independent manner. A three-dimensional model of human TIP30 indicated that the TIP30 protein contains a α-helix protein docking domain near its amino terminus [48], suggesting that TIP30 acts as a tumor suppressor by interacting with other proteins. TIP30 interacts with Importin, a protein located on nuclear membrane, and inhibits the nuclear translocation of a variety of transcription factors [49]. TIP30 negatively regulates the differentiation of oligodendrocytes by inhibiting the level of nuclear Olig1 [50]. In cancer, TIP30 promotes the migration and invasion of cancer cells by suppressing the nuclear translocation of Snail, an epithelial-to-mesenchymal transition inducer [6,19]. Moreover, TIP30 directly binds to P53 and facilitates tumor development [8]. TIP30 retards the endocytic downregulation of EGFR protein, leading to the enhancement of downstream Akt signaling [43]. Consistent with the results from these mechanistic studies, our data supported the finding that the downregulation of the TIP30 protein was associated with a poor NSCLC prognosis.

The clinical value of the TIP30 protein to predict the prognosis of other cancers has been demonstrated by a meta-analysis [51]. Lower TIP30 protein predicted worse relapse-free survival and/or overall survival in esophageal carcinoma, laryngeal carcinoma, glioma, pancreatic carcinoma, breast cancer, gastric cancer, hepatocellular carcinoma and gallbladder carcinoma. Thus far, the association between TIP30 protein levels and the prognosis of NSCLC patients has not yet been determined. Only one study indicated that the TIP30 protein levels decreased in the primary tumors of lung cancer compared to its expression in the adjacent normal tissues, and its expression was even lower in metastatic tumors compared to its levels in primary lesions [29]. From a cohort of 113 NSCLC patients, we showed that the down-regulation of TIP30 was associated with worse progression-free survival and overall survival in NSCLC patients. It is worth noting that, the histological types and disease status of these NSCLC patients are diversified. Most of the histologic types in our cohort were adenocarcinoma (54%, *n* = 61), followed by squamous cell carcinoma (38%, *n* = 43) and large cell carcinoma (8%, *n* = 9). Besides, this cohort contained 43 patients at early stage (stage I and II) and 70 patients at advanced stage (stage III and IV). Future studies are required to clarify the prognostic role of TIP30 protein in each specific subgroup of NSCLC patients. To our knowledge, this is the first follow-up clinical cohort study to validate the prognostic role of TIP30 protein abundance in NSCLC patients. 

In conclusion, we demonstrated that the protein expression, but not the TIP30 mRNA level, was associated with the prognosis of NSCLC patients. A low TIP30 protein expression level was associated with a poor NSCLC prognosis by a clinical follow-up cohort. Our study suggested that the TIP30 protein expression level can be a reliable diagnostic biomarker for NSCLC, thereby enlightening future studies targeting TIP30 in NSCLC cancer treatment.

## Figures and Tables

**Table 1:** Association of TIP30 expression and relapse-free survival in lung cancer patients from the PrognoScan database.

Dataset	Contributor	Array Type	Probe ID	*N*	ln(HR)	HR (95% CI-Low CI-Upp)	Ref
GSE31210	Okayama	HG-U133_Plus_2	229102_at	204	0.065749	1.07 [0.67–1.71]	[30]
			209448_at	204	0.771746	2.16 [1.20–3.90]	
			210253_at	204	0.035683	1.04 [0.62–1.72]	
			207180_s_at	204	0.678586	1.97 [1.26–3.08]	
GSE8894	Lee	HG-U133_Plus_2	210253_at	138	−0.04662	0.95 [0.76–1.19]	[33]
			209448_at	138	0.191915	1.21 [0.93–1.57]	
			207180_s_at	138	0.179512	1.20 [0.96–1.49]	
			229102_at	138	−17.9383	0.00 [0.00–0.19]	
GSE17710	Wilkerson	Agilent-UNC-custom-4X44K	10368	56	−0.22019	0.80 [0.51–1.27]	[34]
			42549	56	−0.10024	0.90 [0.61–1.34]	
			11172	56	−0.10444	0.90 [0.60–1.35]	

Abbreviations: HR, hazard ratio; Low, lower; Upp, upper; Ref, reference.

**Table 2:** Association of TIP30 expression and overall survival in lung cancer patients from the PrognoScan database.

Dataset	Contributor	Array Type	Probe ID	*N*	ln(HR)	HR (95% CI-Low CI-Upp)	Ref
jacob-00182-CANDF	Shedden	HG-U133A	209448_at	82	0.433604	1.54 [0.70–3.38]	[35]
			210253_at	82	1.03816	2.82 [0.90–8.86]	
			207180_s_at	82	0.670805	1.96 [1.00–3.81]	
HARVARD-LC	Beer	HG-U95A	38824_at	84	0.406418	1.50 [0.93–2.42]	[36]
jacob-00182-HLM	Shedden	HG-U133A	209448_at	79	0.212509	1.24 [0.70–2.18]	[35]
			207180_s_at	79	0.277452	1.32 [0.85–2.06]	
			210253_at	79	0.176389	1.19 [0.62–2.28]	
jacob-00182-MSK	Shedden	HG-U133A	207180_s_at	104	−0.13325	0.88 [0.51–1.51]	[35]
			209448_at	104	−0.28405	0.75 [0.36–1.55]	
			210253_at	104	−0.25751	0.77 [0.32–1.89]	
GSE13213	Tomida	G4112F	A_23_P64129	117	0.464958	1.59 [1.01–2.50]	[37]
			A_24_P307580	117	0.080112	1.08 [0.78–1.50]	
GSE31210	Okayama	HG-U133_Plus_2	229102_at	204	0.288019	1.33 [0.73–2.45]	[30]
			209448_at	204	1.28166	3.60 [1.70–7.63]	
			210253_at	204	0.54017	1.72 [0.93–3.18]	
			207180_s_at	204	0.947811	2.58 [1.51–4.41]	
jacob-00182-UM	Shedden	HG-U133A	209448_at	178	−0.06997	0.93 [0.56–1.55]	[35]
			210253_at	178	0.170614	1.19 [0.69–2.03]	
			207180_s_at	178	0.101831	1.11 [0.74–1.66]	
GSE11117	Baty	Novachip human 34.5k	H200010450	41	−0.06557	0.94 [0.59–1.48]	[24]
GSE3141	Bild	HG-U133_Plus_2	209448_at	111	0.072535	1.08 [0.71–1.62]	[38]
			210253_at	111	−0.1588	0.85 [0.53–1.38]	
			207180_s_at	111	0.02155	1.02 [0.73–1.44]	
			229102_at	111	0.001905	1.00 [0.69–1.46]	
GSE14814	Zhu	HG-U133A	209448_at	90	0.13546	1.15 [0.71–1.85]	[25]
			210253_at	90	0.445921	1.56 [0.53–4.59]	
			207180_s_at	90	0.209906	1.23 [0.66–2.29]	
GSE4716-GPL3694	Tomida	GF200	2775	50	0.41877	1.52 [0.60–3.87]	[39]
GSE4573	Raponi	HG-U133A	207180_s_at	129	0.445355	1.56 [0.76–3.20]	[40]
			209448_at	129	0.183704	1.20 [0.72–2.00]	
			210253_at	129	0.021825	1.02 [0.57–1.84]	
GSE17710	Wilkerson	Agilent-UNC-custom-4X44K	42549	56	0.056871	1.06 [0.70–1.60]	[34]
			11172	56	0.055057	1.06 [0.69–1.61]	
			10368	56	−0.01173	0.99 [0.62–1.57]	

Abbreviations: HR, hazard ratio; Low, lower; Upp, upper; Ref, reference.

**Table 3:** Demographics and characteristics of the patients and the association of TIP30 expression and clinicopathological parameters.

	TIP30 Expression		
Characteristics	Low (0, 1) (*n* = 67)	High (2, 3) (*n* = 46)	*P*-Value
Age			0.962 ^†^
Years (mean ± SD)	62.10 ± 10.06	62.20 ± 9.85	
Gender			0.498 ^‡^
Male	35	27	
Female	32	19	
Stage			0.013 ^‡^*
I	14	16	
II	4	9	
III	21	12	
IV	28	9	
Tumor status			0.929 ^‡^
T1	11	9	
T2	32	23	
T3	4	2	
T4	20	12	
Lymph node status			0.059 ^‡^
N0	19	21	
N1–3	48	25	
Distal metastasis status			0.027 ^‡^*
M0	39	36	
M1	28	10	
Histologic type			0.016 ^‡^*
Adenocarcinoma	31	30	
Squamous cell	27	16	
Large cell	9	0	
Recurrence			0.002 ^‡^*
No	27	32	
Yes	40	14	
Smoking status			0.093 ^‡^
No	34	16	
Yes	33	30	

* *P* < 0.05 was considered statistically significant. ^†^ Student’s *t* test for continuous variables. ^‡^ Pearson’s chi-square test for categorical variables.

**Table 4:** Cox univariate and multivariate regression analysis of prognostic factors for overall and progression-free survival in 113 NSCLC patients.

**Cox univariate analysis (OS)**			
Variables	Comparison	HR (95% CI)	*P*-value
T	T1; T2−T4	2.099 (1.157−3.807)	0.015 *
N	N0; N1−N3	2.803 (1.705−4.608)	<0.001 *
M	M0; M1	2.952 (1.881−4.632)	<0.001 *
TIP30	Low (0, 1); High (2, 3)	0.410 (0.252−0.667)	<0.001 *
**Cox multivariate analysis (OS)**			
Variables	Comparison	HR (95% CI)	*P*-value
T	T1; T2−T4	1.603 (0.879−2.924)	0.124
N	N0; N1−N3	2.061 (1.240−3.424)	0.005 *
M	M0; M1	2.121 (1.338−3.361)	0.001 *
TIP30	Low (0, 1); High (2, 3)	0.550 (0.333−0.909)	0.020 *
**Cox univariate analysis (PFS)**			
Variables	Comparison	HR (95% CI)	*P*-value
T	T1; T2−T4	1.608 (0.781−3.313)	0.198
N	N0; N1−N3	3.238 (1.668−6.286)	0.001 *
M	M0; M1	2.525 (1.453−4.387)	0.001 *
TIP30	Low (0, 1); High (2, 3)	0.384 (0.206−0.715)	0.003 *
**Cox multivariate analysis (PFS)**			
Variables	Comparison	HR (95% CI)	*P*-value
T	T1; T2−T4	1.148 (0.555−2.378)	0.709
N	N0; N1−N3	2.634 (1.340−5.176)	0.005 *
M	M0; M1	1.810 (1.028−3.187)	0.040 *
TIP30	Low (0, 1); High (2, 3)	0.513 (0.271−0.970)	0.040 *

* *P* < 0.05 was considered statistically significant. PFS: progression free survival. OS: over-all survival.
